# BMI Was Maintained Among Women with Low Incomes in Indiana Who Participated in Food Assistance and/or Federal Nutrition Education Over 1 Year

**DOI:** 10.1016/j.cdnut.2026.107651

**Published:** 2026-01-30

**Authors:** Yue Qin, Bruce A Craig, Regan L Bailey, Angela R Abbott, Blake A Connolly, Heather A Eicher-Miller

**Affiliations:** 1 Department of Nutrition Science, Purdue University, West Lafayette, IN, United States; 2 Department of Statistics, Purdue University, West Lafayette, IN, United States; 3 Department of Nutrition, Institute for Advancing Health through Agriculture, Texas A&M University, College Station, TX, United States; 4 Health and Human Sciences Extension, Purdue University, West Lafayette, IN, United States

**Keywords:** obesity, body mass index, nutrition education, food assistance, SNAP-Ed, low-income, federal programs

## Abstract

**Background:**

Longer-term (≥1 y) associations between federal food assistance and federal nutrition education with BMI are unclear.

**Objectives:**

This exploratory study determined the relationship of participation in a federal nutrition education intervention and self-selected food assistance program participation, and their combination on BMI for ≥1 y.

**Methods:**

Women (≥18 y) from Indiana, the United States were experimentally assigned to receive federal nutrition education of the United States (US) Supplemental Nutrition Assistance Program-Education (SNAP-Ed) program, including the 4 lessons fulfilling SNAP-Ed guidance (*n* = 59) or not (*n* = 47), or to a control group, in this longitudinal study from August 2015 till May 2017 (registered at www.clinicaltrials.gov as NCT03436784). Food assistance participation was self-selected. Main outcomes were averaged triplicate measured height and weight for BMI at baseline and 1-y by trained paraprofessionals. Analysis included mixed linear models comparing BMI over time by participation in the intervention or not, food assistance or not, or combinations of both programs.

**Results:**

Obesity was classified for most participants (>60%) at both time points. BMI did not differ, nor were differences observed in BMI change >1 y based on receiving SNAP-Ed, food assistance programs, or their combination.

**Conclusions:**

Weight was constant >1 y regardless of receiving federal nutrition education, food assistance programs, or their combination, suggesting neither these programs nor their combination cause weight gain among low-income US women in this preliminary study of these interventions among this sample.

## Introduction

Overweight and obesity are prevalent health risks in the United States. Based on the latest available national data from 2024, 4 of 10 adults were classified as obese [1], a 10% increase compared with 2 decades prior [2]. This high prevalence of obesity is of public health concern, as obesity is associated with numerous chronic diseases, including but not limited to diabetes mellitus, cardiovascular disease, nonalcoholic fatty liver disease, hypertension, and dyslipidemia [3]. Increased risk of obesity is linked with low-income [4] as well as food insecurity, defined as having inadequate access to food for active and healthy lives [5], especially among adult women [6]. Food insecurity is also associated with poor diet quality, which is a modifiable risk factor for obesity and weight management [7,8].

To improve the food access and security of households with low-incomes, the USDA Food and Nutrition Service provides food assistance through several programs, including the Supplemental Nutrition Assistance Program (SNAP) and the Special Supplemental Nutrition Program for Women, Infants, and Children (WIC). SNAP, previously known as the food stamp program, is aimed to alleviate food insecurity by improving access to healthy foods through a monetary benefit to US households that qualify (household income ≤130% of the federal poverty line or poverty income ratio[ PIR]) [9]. The federal nutrition education program Supplemental Nutrition Assistance Program-Education (SNAP-Ed) compliments SNAP and provides nutrition education to adults eligible for SNAP [10], promoting a healthy lifestyle and behaviors aligned with the Dietary Guidelines for Americans [11]. The federally funded nutrition assistance program WIC provides monthly benefits to pregnant, breastfeeding, or nonbreastfeeding postpartum women, infants, toddlers, and children <5 y within households with gross incomes ≤185% of the PIR to purchase certain supplemental foods with the goal of improving food access and nutrition [12]. WIC food packages were revised in 2009 to promote closer alignment to updated nutrition and infant feeding guidelines[13].

Despite these programs, living in situations of few resources presents risks for poor nutrition and health and the food assistance eligible population experience a high burden of poor dietary intakes and chronic conditions [7,[14], [15], [16]]. Weight is a major risk factor for health and a relevant longer-term outcome for evaluating SNAP, SNAP-Ed, and WIC because of their goals to promote food access, nutrition and health. The programs are hypothesized to ameliorate food and nutrition security risks and to support healthy lifestyles. However, since SNAP and WIC provide supplemental food, they are often criticized for contributing to obesity prevalence [17]. Scientific evidence of causal links of program effects on health outcomes is difficult to establish because individuals that use these programs may already be in worse financial and health situations than those who do not use these programs. Program resources provided through SNAP and WIC may also not be ample to support health or dietary improvements and their use may be hindered by the food available in the environment. However, these programs may potentially prevent further declines in nutrition and health. The relevance of nutrition education to healthy weight is addressed through core topics that federal guidance on SNAP-Ed outlines including energy balance and selection of nutrient-dense foods without excess energy on a limited budget, using the Nutrition Facts label, choosing from varied and healthful food groups such as whole grains, fruits, and vegetables according to the Dietary Guidelines for Americans, and promoting active lifestyles [10,11].

Another difficulty in providing evidence of program effects is the designation of SNAP as an entitlement program, preventing ethical withholding of the program to create control groups. Cross-sectional or longitudinal cohort study designs have been used to evaluate SNAP, SNAP-Ed, and WIC programs use with food security, diet, and weight as outcomes in the scientific literature and show stronger results for linkage with food security compared with inconsistent evidence on diet and weight. For example, SNAP participation improves food security status [18], but mixed results have been shown for diet quality [19,20] and weight status [[21], [22], [23], [24]]. Cross-sectional and longitudinal studies have also shown a higher likelihood of weight gain and obesity among SNAP participants, especially for women [[21], [22], [23]], whereas another study did not find associations between weight gain and obesity with shorter or longer-term SNAP participation [24].

SNAP-Ed, in contrast to SNAP, is a nonentitlement program, allowing stronger evidence resulting from experimental controlled study designs that have shown improved food security >1 y later due to SNAP-Ed [25,26]. SNAP-Ed adult participants were more likely to consume fruits and vegetables after nutrition education compared with before in an 8-state study [27,28], and a review showed SNAP-Ed improved nutrition-related behaviors [27], yet another study showed that only vitamin D out of several dietary outcomes improved [15]. A high prevalence of obesity was noted among SNAP-Ed participants in these studies [29,30,]], but the impact of SNAP-Ed over a longer period of 1 year on weight status was not evaluated despite program goals to promote healthy lifestyles, including dietary intake, according to the Dietary Guidelines for Americans, and physical activity [10,11]. Evidence for the nonentitlement program WIC is also limited. On one hand, some studies found improvements in certain dietary outcomes, including diet quality and nutrient intake after the WIC food package revision [31,32]. However, evaluation of WIC participation and weight status among adult participants is missing and potentially challenging to determine due to the specificity of WIC eligibility, including populations that might undergo frequent weight changes throughout pregnancy and lactation. Therefore, knowledge of the impact of SNAP-Ed, SNAP, and/or WIC over time on weight status among SNAP-Ed participants remains unclear. Notably, a large proportion of households with low-incomes (∼73%) used food assistance programs together with additional programs like nutrition education and WIC [25,33,34], yet the combination of these programs on weight status has not been evaluated. A study on the programs relationship to weight status should consider the realistic situation of both single and multiple program utilization and combinations of use of all 3 programs. An experimental SNAP-Ed study design that accounts for self-selected use of SNAP and WIC facilitates the evaluation of real-life utilization and the relationship to weight status to determine whether program goals of promoting health and weight status maintenance or alternatively, causing weight gain, would advance knowledge on these relationships.

To address these gaps, the present investigation was carried out to determine the potential for association between an exposure of 1-y participation of food assistance and/or nutrition education programs with weight status by BMI as the outcome, specifically to evaluate the comparisons of: *1*) experimentally assigned SNAP-Ed intervention compared with control; *2*) self-selected SNAP participation compared with no SNAP participation; *3*) SNAP participation only compared with both SNAP and self-selected WIC participation; and *4*) both a SNAP-Ed intervention and SNAP and/or WIC compared with SNAP and/or WIC (no SNAP-Ed intervention) or a SNAP-Ed intervention only (neither SNAP nor WIC), respectively. To best of our knowledge, this was the first attempt to examine the association of single or multiple program participation in SNAP, WIC, and SNAP-Ed with weight status on nonpregnant, adult low-income women, and weight status is therefore considered exploratory.

## Methods

### Study population and design

The present study was a secondary analysis of data from the Indiana SNAP-Ed 1-y Study, a longitudinal, controlled trial with a direct nutrition education intervention where a series of ≥4 SNAP-Ed educational lessons were provided directly to adults to determine an effect on dietary intake; complete details of the study can be found elsewhere [15]. Briefly, participants were recruited from Indiana by county-level SNAP-Ed nutrition educators from Purdue University’s Health and Human Science Cooperative Extension Nutrition Education Program. SNAP-Ed paraprofessionals screened potential participants and assigned eligible participants into intervention (i.e., immediate lessons) and control or delayed lessons (1 y later) groups at a ratio of around 1:1. Participants who were eligible for SNAP-Ed [10], did not receive SNAP-Ed lessons in the year prior to recruitment, able to read English, Indiana residents, and willing to wait for 1 y to receive the nutrition lessons, were recruited. A random number generator was used to assign the first participant (or group) into the intervention or control group to prevent knowledge of treatment assignment. Subsequent participants were assigned to alternating treatment groups. Participants were compensated with ≤ $30 and $35 Walmart gift cards for completing all baseline and follow-up assessments, respectively. All participants provided written informed consent, and the Purdue University’s Institutional Review Board approved the trial protocol, which was registered at www.clinicaltrials.gov as NCT03436784, where the primary endpoint was dietary intake with a secondary outcome of nutrient intake that did not change during the course of the study (except for vitamin D) or in post-hoc analysis. The current study used data on heights and weights to carry out an evaluation of a BMI endpoint.

### SNAP-Ed intervention

The intervention was planned according to SNAP-Ed Plan Guidance to consist of ≥4 core nutrition education lessons (of 10) in the “Small Steps to Health” Indiana SNAP-Ed curriculum [15], addressing USDA key behavioral outcomes. Specifically, the 4 required lessons fulfilled the federal guidance for the program and encouraged use of USDA MyPlate [35] and food labels to build energy-balanced diets, highlighted the importance of whole grains, fruits, and vegetables, and encouraged incorporation of these food groups to make nutrient-dense food choices and incorporating daily physical activity [10,11]. The SNAP-Ed curriculum also included elements that helped participants develop resource management skills, such as thrifty meal-planning and food-dollar budgeting [10,11]. Additional lessons [[5], [6], [7], [8], [9], [10]] were allowed but not required. The lessons were delivered directly and inperson to participants by paraprofessionals, in 1-on-1 or group settings and the paraprofessionals verified participation in each of the lessons.

### Measures

All participants completed a baseline assessment with a basic characteristic questionnaire including sociodemographic variables, and food assistance program use at recruitment from August 2015 to May, and at 1 y follow-up, from August 2016 to May 2017. SNAP and WIC use were collected with questions “Do you currently receive food stamps or SNAP” and “Do you currently receive WIC benefits?” at both baseline and 1 y follow-up. The SNAP-Ed paraprofessionals were trained to measure height and weight using protocols developed by the National Health and Nutrition Examination Survey [36] at both baseline and follow-up assessments. At each assessment, height and weight were each measured 3 separate times. The mean heights and weights for each participant at each timepoint were calculated and used to determine BMI at baseline and at follow-up. BMI was categorized into underweight (BMI <18.50 kg/m^2^), normal (18.5 ≤ BMI <25 kg/m^2^), overweight (25 ≤BMI <30 kg/m^2^), and obese (BMI ≥30 kg/m^2^) groups [37].

### Statistical analysis

A total of 106 participants (*n* = 47 control and *n* = 59 intervention) were included in the analysis (Figure). Participants were excluded from analysis (*n* = 6 control and *n* = 15 intervention) for incomplete data on SNAP-Ed, SNAP, and/or WIC (*n* = 3), being male (*n* = 6) due to the small number, and pregnancy at any time during the study (*n* = 8) due to inherent BMI changes. Chi-square tests were used to compare sociodemographic characteristics for each of the comparison groups to identify potential confounding variables to adjust in the models, and to determine differences between those who completed the study and those who withdrew early, and to compare study completers with a limited set of characteristics of the Indiana SNAP-Ed population to allow comment on generalizability of the results. Mixed linear regression models, using BMI as the response, were used to compare changes in BMI from baseline to 1 y follow-up across the various comparison groups. Several covariates were included in the models with group, time, and their interaction as fixed effects and participant as a random effect. Educator assignment was checked and did not differ between groups; thus was not adjusted in the model. Change of food assistance program status >1 y was identified as a factor that may have a link to the BMI comparison of food assistance program participants compared with nonparticipants. A variable was constructed to account for this factor using the self-reported SNAP and WIC status at baseline and follow-up assessments and categorized as “no change,” “changed out of the program” and “changed into the program” for SNAP and WIC respectively, and this variable was included in comparisons 2 (SNAP participants compared with nonparticipants), 3 (SNAP only compared with SNAP and WIC participants), and 4 (SNAP-Ed and SNAP and/or WIC compared with SNAP and/or WIC only or SNAP-Ed only). A small number of participants (*n* = 22 for SNAP, *n* = 13 for WIC, or ∼1/3 of each group) had changes in SNAP and/or WIC program participation status over the 1 y of the study, and a sensitivity analysis where these participants were removed from the analysis was carried out but the linear regression results did not change. Therefore, the participants were retained for the final analysis. In addition, an adjustment for the SNAP-Ed treatment group assignment was made in comparisons 2, 3, and 4. Chi-square tests indicated no differences between the comparison 1, SNAP-Ed intervention compared with comparison groups (Table 1). Thus, only changes of SNAP and WIC use over time were adjusted in comparison 1. A few characteristics were different for the other comparisons 2 to 4 (Supplementary Tables 1-3). All characteristics (those different or not different between comparison groups) were examined in the model and those that contributed to predictivity were included as covariates in their final respective models. Specifically, age was adjusted in comparison 2. The power to detect a difference at significance level of 0.05 of *n* = 106 exceeded that needed for power at 80% based on mean 1.5 to 2.0 BMI difference between SNAP-Ed intervention and control groups >1 y, relating to a 3% to 5% clinically meaningful decrease [38,39]. This BMI decrease was achieved in a previous study among *n* = 93 low-income women with a high prevalence of overweight and obesity as in this study. Women in the prior study also received a lifestyle educational intervention with similar components as included in the SNAP-Ed intervention of the present study, yet the present study is regarded as exploratory because the sample and intervention are somewhat unique from the prior study [40]. All analyses were performed using SAS software, version 9.4 [41].

**FIGURE:**
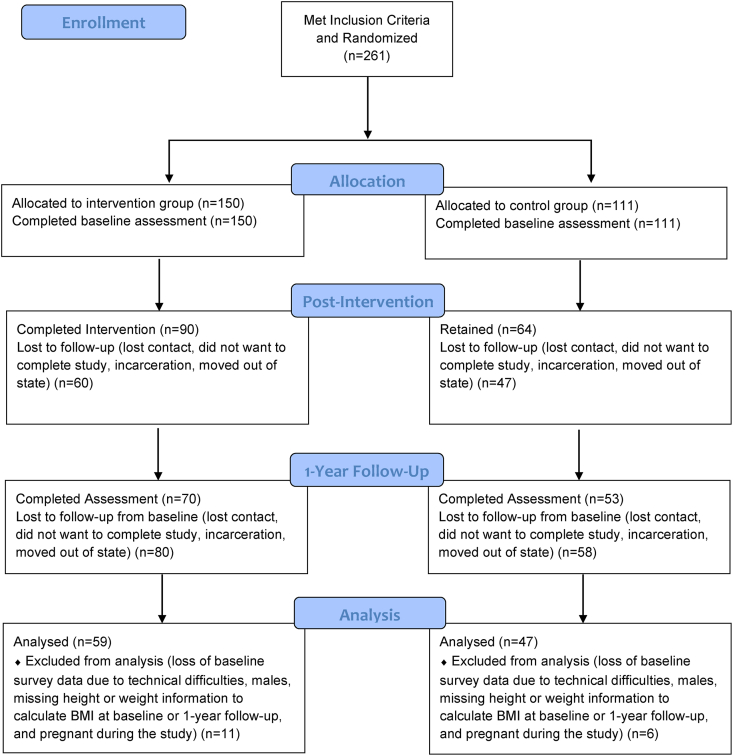
Participant flow chart for loss to follow-up and assessment completion among adult women Indiana Supplemental Nutrition Assistance Program-Education eligible participants from 2015 to 2017.

**TABLE 1:** Baseline sociodemographic characteristics by Supplemental Nutrition Assistance Program-Education (SNAP-Ed) intervention or control group and BMI status at baseline and 1 y follow-up among adult Indiana SNAP-Ed-eligible female participants from 2015 to 2017^1^

Characteristics	Categories	Control		SNAP-Ed Intervention		*P* value
		*n*	%	*n*	%	
Total (*n* = 106)	—	47	46	59	54	—
Age group (y)	—	—	—	—	—	0.20
	18–30	18	38	19	32	—
	31–50	21	45	21	36	—
	≥51	8	17	19	32	—
Race and Ethnicity	—	—	—	—	—	0.76
	Non-Hispanic white	43	91	54	93	—
	Other	4	9	4	7	—
Household education	—	—	—	—	—	—
	No high school diploma	12	26	8	14	0.24
	HS diploma/GED	13	28	24	41	—
	Some college	14	30	16	27	—
	Associate degree	4	9	9	15	—
	Bachelor’s degree or more	4	9	2	3	
Marital status	—	—	—	—	—	0.66
	Never married	11	23	10	17	—
	Married w/ partner	22	47	28	47	—
	Separated/divorced/widowed	14	30	21	36	—
No. of other household adults	—	—	—	—	—	0.08
	None	6	13	18	31	—
	1 Additional	21	45	20	34	—
	2 Additional	8	17	13	22	—
	3 or more additional	12	26	8	14	—
No. of household children	—	—	—	—	—	0.49
	0	12	26	17	29	—
	1–2	18	38	27	46	—
	≥3	17	36	15	25	—
Employed in last 12 mo	—	—	—	—	—	0.91
	No	26	55	32	54	—
	Yes	21	45	27	46	—
	Part-Time	12	60	12	44	0.29
	Full-Time	8	40	15	56	—
Other household adult employment in the last 12 mo	—	—	—	—	—	0.29
	No	19	40	29	51	—
	Yes	28	60	28	49	—
	Part-time	7	26	6	22	0.75
	Full-time	20	74	21	78	—
Monthly income ($)	—	—	—	—	—	0.29
	0–1265	12	27	25	42	—
	1266–1705	12	27	9	15	—
	1706–2144	7	16	10	17	—
	≥2145	14	31	15	25	—
SNAP^2^ participation	—	—	—	—	—	0.60
	Yes	31	66	36	61	—
	No	16	34	23	39	—
WIC^3^	—	—	—	—	—	0.74
	Yes	19	40	22	37	
	No	28	60	37	63	
Emergency food assistance participation (food pantry)	—	—	—	—	—	0.21
	No	26	55	25	41	—
	Yes	21	45	33	59	—
	<1 per mo	5	24	3	9	0.50
	1 time per mo	10	48	18	55	—
	1–3 times a mo	5	24	9	27	—
	≥1 per wk	1	5	3	9	—
BMI at baseline (kg/m^2^)						
	Underweight	2	4	0	0	—
	Normal	7	15	9	16	—
	Overweight	8	17	13	22	—
	Obese	30	64	35	61	—
BMI at follow-up (kg/m^2^)						
	Underweight	2	4	0	0	—
	Normal	9	19	7	12	—
	Overweight	7	15	16	28	—
	Obese	29	62	34	60	—

Abbreviations: GED, General Educational Development Test; SNAP, Supplemental Nutrition Assistance Program; WIC, Special Supplemental Nutrition Program for Women, Infants, and Children

1 Data were number of participants and percent. Chi-Square tests were used to compare the characteristics. Statistical significance at P < 0.05. All data were self-reported. Total numbers do not always add up to sample size because of missing values; percentages do not always add up to 100 because of rounding.

2 SNAP participation reference time period was the previous 30 d.

3 WIC, participation reference time period was the previous 30 d.

## Results

The sample was predominately non-Hispanic White women. A majority of the sample were >30 y old, with educational attainment below bachelor’s degree, living in households with ≥ 1 other adult and with children for both intervention and control groups (Table 1). The sample differed from the participants who withdrew from the study in age, marital status, employment in the last 12 mo, and WIC participation (Supplementary Table 4). The study sample was different from the greater Indiana SNAP-Ed client population in SNAP and WIC participation, race and ethnicity, sex, age, and number of household children (data not shown).

BMI was high (both SNAP-Ed intervention and control group) at each time point, with >60% of the group classified as having obesity (Table 1). Mean BMI for all comparison groups, ranging from 28.4 to 40.9 kg/m^2^, was classified as obese or overweight (Table 2). There were no significant differences in change of BMI over time between the comparisons 1 (SNAP-Ed intervention group compared with control group), 2 (SNAP participants compared with nonparticipants), 3 (SNAP only participants compared with SNAP and WIC participants), or 4 (SNAP-Ed and SNAP and/or WIC compared with SNAP and/or WIC only or SNAP-Ed only) (Table 2).

**TABLE 2:** One-year difference in changes in BMI between nutrition education through Supplemental Nutrition Assistance Program-Education (SNAP-Ed) intervention and control groups; food assistance through the SNAP self-assigned participants and nonparticipants; and combinations of participating in one or multiple nutrition education or food assistance programs among adult women SNAP-Ed-eligible study participants

Comparison 1	Control (*n* = 47)				SNAP-Ed intervention (*n* = 59)				Difference in changes^1^			
	Baseline		1-y follow-up		Baseline		1-y follow-up		ΔSNAP-Ed Intervention – Δ Control^2^		*P* value^3^	95% CI^4^
	LSM	SE	LSM	SE	LSM	SE	LSM	SE	LSM	SE		
BMI (kg/m^2^)	32.9	7.7	32.6	7.7	38.7	6.9	37.3	6.9	−1.0	0.9	0.23	−2.8 to 0.8

Comparison 2	SNAP participants (*n* = 67)				SNAP nonparticipants (*n* = 39)				Difference in changes^5^			

	Baseline		1-y follow-up		Baseline		1-y follow-up		ΔSNAP participants – Δ SNAP nonparticipants^2^		*P* value^3^	95% CI^4^
LSM	SE	LSM	SE	LSM	SE	LSM	SE	LSM	SE			
BMI (kg/m^2^)	40.4	7.8	40.0	7.7	35.9	7.8	33.9	7.8	−1.7	0.9	0.07	−3.5 to 0.1

Comparison 3	SNAP only (*n* = 38)				Both SNAP and WIC (*n* = 29)				Difference in Changes^6^			

	Baseline		1-year follow-up		Baseline		1-y follow-up		ΔSNAP only - Δ both SNAP and WIC^2^		*P* value^3^	95% CI^4^
LSM	SE	LSM	SE	LSM	SE	LSM	SE	LSM	SE			
BMI (kg/m^2^)	40.9	2.8	40.4	2.8	38.4	2.7	38.1	2.6	0.2	1.1	0.83	−2.0 to 2.4

Comparison 4	Group 1 (*n* = 41)^7^				Group 2 (*n* = 65)^8^				Difference in changes^9^			

	Baseline		1-y follow-up		Baseline		1-y follow-up		Δ Group 1- Δ group 2^2^		*P* value^3^	95% CI^4^
LSM	SE	LSM	SE	LSM	SE	LSM	SE	LSM	SE			
BMI (kg/m^2^)	29.2	9.0	28.4	9.0	38.0	7.1	37.0	7.1	−0.2	0.9	0.85	−2.0 to 1.6

Abbreviation: LSM, least square means.

1 Outcomes were controlled for changes in Supplemental Nutrition Assistance Program (SNAP) and Special Supplemental Nutrition Program for Women, Infants, and Children (WIC) participation over time. Difference in changes were mean change of BMI in control group from 1-y follow-up and baseline subtracted from the mean change in intervention group at 1-y follow-up and baseline.

2 Δ, difference in changes.

3 Significance level was P < 0.05.

4 95% confidence intervals were for difference in changes.

5 Both groups contained participants in SNAP-Ed intervention and control groups. Outcomes were controlled for SNAP-Ed treatment group assignment, change in SNAP status over time (no change, changed out of SNAP or changed into SNAP), change in Special Supplemental Nutrition Program for Women, Infants, and Children (WIC) status over time (no change, changed out of WIC or changed into WIC) and age. Difference in changes were mean change of BMI among SNAP participants from 1-year follow-up and baseline subtracted from the mean change among SNAP nonparticipants at 1-year follow-up and baseline.

6 Outcomes were controlled for SNAP-Ed treatment group assignment and change in SNAP status over time (no change, changed out of SNAP/WIC or changed into SNAP/WIC). Difference in changes were mean change of BMI among SNAP only participants from 1-y follow-up and baseline subtracted from the mean change among SNAP and WIC participants at 1-y follow-up and baseline.

7 Group 1: SNAP-Ed with SNAP and/or WIC.

8 Group 2: no SNAP-Ed with SNAP and/or WIC; or SNAP-Ed with neither SNAP nor WIC.

9 Outcomes were controlled for SNAP-Ed treatment group assignment and change in SNAP status to 1-y follow-up (no change, changed out of SNAP/WIC or changed into SNAP/WIC). Difference in changes were mean change of BMI among SNAP only participants from 1-y follow-up and baseline subtracted from the mean change among SNAP and WIC participants at 1-y follow-up and baseline.

## Discussion

The results of this exploratory study show no difference in weight status changes >1 y for any of the comparisons featured among this group of women with low incomes. The weight maintenance over time supports that food assistance program use, such as SNAP, WIC, and/or SNAP-Ed nutrition education, is not giving rise to weight gain in this sample of low-income women in Indiana [[21], [22], [23],^34^].

Comparison 1 focused on the experimentally assigned SNAP-Ed. The Indiana SNAP-Ed curriculum delivered through the intervention includes lessons that focus on the promotion of healthful lifestyles through energy-balanced dietary choices and regular physical activity [10,42]. To this end, the exploratory results here show the participants did not gain weight over 1 y [15]. Previous literature suggests women of low-income who participated in food assistance programs were up to 10% more likely to be obese and gain weight (1.24 unit increase in BMI) over time [21,[43], [44], [45]]. Thus, being able to maintain weight status over time supports that SNAP-Ed did not increase risk of obesity over time among SNAP-Ed participants. Among the SNAP-Ed intervention group, there was a 1.4 kg/m^2^ decrease in BMI >1 y that was not statistically significant paired with the means ± SD in our sample, yet the estimate may potentially be meaningful to other health outcomes, such as blood pressure and lipid profile and should be evaluated in future studies [46,47]. The decrease was smaller than expected based on a former study using a similar intervention, where *n* = 93 [40], of which estimates may have potentially been on the higher end of an effect size spectrum. Using the results from our specific sample, a sample of >700 may power a future study and could be helpful in estimations to evaluate federal nutrition education interventions. This sample size might not be feasible without substantial financial support, labor resources, and cross-state or nation-wide collaborations. With a larger sample, a comparison of the BMI changes over time for those with change in participation status for SNAP and/or WIC compared with those whose participation status did not change over time could be an additional valuable aspect to consider.

Similarly, in comparison 2, where BMI changes over time were contrasted between SNAP participants and nonparticipants (self-assigned), no weight status differences were detected. In contrast, previous literature [[21], [22], [23],^34^] has shown current and long-term participation in SNAP was associated with weight gain and obesity. Like comparison 1, a statistically nonsignificant estimate of lower BMI (2.0 kg/m^2^) was observed over time for SNAP nonparticipants but not for SNAP participants, with a potential relevance to health [46,47]. Different from SNAP-Ed, SNAP was not experimentally allocated between the comparison groups, so there may be unaccounted for differences between the SNAP comparison groups [41].

Comparison 3 featured those using only SNAP compared with those who used both SNAP and WIC (self-assigned) over time and showed no differences in BMI changes over time. Although there are no prior studies that examined WIC participation with weight status, evidence has shown WIC participation was linked to improved nutrient intake and diet quality among women [31,32], which could help maintain a healthy weight over time, whereas participation in SNAP in some studies has been associated with increased weight [[21], [22], [23],^34^]. The present results suggest that participating in either or both programs were not associated with weight status changes over time.

In comparison 4, the authors evaluated the unique combination of SNAP-Ed and SNAP and/or WIC compared with those that were in SNAP and/or WIC, or SNAP-Ed only. No prior studies have assessed the combination of SNAP and SNAP-Ed on weight status among participants, highlighting a gap that this study fills. The 2 programs are designed to complement each other with the aim to improve both the access and quality of food, and ultimately health, among groups with low-incomes. This study was a first step to future research that may explore various combinations of food assistance and nutrition education participation, such as separate evaluation of groups by food assistance only, food assistance and nutrition education, nutrition education only, and neither food assistance nor nutrition education, on BMI to evaluate and identify the potentially synergistic effects of these programs on weight status outcomes. Since different groups of individuals might respond differently to different programs and have differential impacts on a spectrum of health outcomes, understanding the potentially interacting effects of programs could help tailor health promotion toward target populations. Additionally, such investigations could inform the possibility to deliver interventions using existing program frameworks in various combinations, which might result in enhanced effectiveness and reduced implementation cost.

The overall results from the present analysis showed high obesity among SNAP-Ed eligible Indiana women (mean BMI >30 kg/m^2^) for all comparison groups, at all time points except for those participating in both education and food assistance. The findings demonstrated higher mean BMI (28.4–40.9 kg/m^2^) and prevalence of obesity (60%–64%) among this sample of Indiana SNAP-Ed-eligible women compared with those reported by a previous study [29] that featured SNAP-Ed participants in Georgia, 30% and 42%, respectively. The previous study [29] included both women and men, sampled from a different state, a larger sample size (*n* = 270), and older population (mean age 60 y) compared with the current study, which might contribute to the differences in the BMI results. The prevalence of obesity from the current sample was also higher compared with that of US women in general (41%) and with low-incomes (42%) [1,48], which indicated higher health risks among the Indiana SNAP-Ed-eligible women featured here.

Limitations of the study are a high attrition rate from the baseline to the follow-up assessments, which is common for longitudinal studies, but which could have influenced the results. Nonetheless, the study used an experimentally controlled design for the primary intervention of SNAP-Ed on BMI. There were differences in certain characteristics between the final analytic sample and those, who did not complete the study, specifically those who withdrew were more likely to be younger, never married, employed in the past 12 mo, and more likely to use WIC compared with study participants. Furthermore, comparison of a limited set of characteristics of the study sample with the Indiana SNAP-Ed client population showed that those in the study differed (compared with the general Indiana SNAP-Ed client population) by SNAP and WIC participation (higher), race, and ethnicity (more non-Hispanic White), sex (all women), age (younger), and number of children in the household (more). Thus, the current findings might not represent those who did not complete all study procedures, nor the Indiana SNAP-Ed population. Future research on this population should explore creative ways to retain participants, include a larger sample size, and obtain a more representative analytic sample [48]. One additional limitation is in the WIC participation status query (“do you currently receive WIC benefits”), which is somewhat ambiguous since participants could have affirmed for their own participation, but they may also have affirmed if they receive WIC on behalf of a child. If the former, it may mean they are <1 y postpartum, a time of potentially dynamic weight status that is independent from program participation and which could have influenced comparisons 3 and 4 but are less likely to have influenced comparisons 1 and 2 (no evidence for significantly different distribution of WIC among groups).

Results of this study provide important insights into weight status by federal food assistance and nutrition education participation, suggesting no evidence of weight gain among a population at risk. The study is an important exploratory step in assessing the complex associations between various assistance and education program use with BMI. The present analysis shows the feasibility of an experimental study design for SNAP-Ed and methodology that was effectively carried out for use in future evaluations. Weight status in this group of Indiana women eligible for SNAP-ED and with a high prevalence of obesity, did not increase over 1 y by receipt of food assistance and a federal nutrition education intervention separately or in combination suggesting preliminary results that these programs may not cause weight gain.

## Author contributions

The authors’ responsibilities were as follows – HE-M: conceptualized the work; YQ, HE-M, and BC: created the methodology; Y.Q: performed formal analysis, wrote the original draft; YQ, BAC, RLB, ARA, BAC, and HE-M: reviewed and edited the script; HE-M: supervised all processes and acquired funding. All authors have read and agreed to the published version of the manuscript.

## Data availability

The data described in the manuscript will not be made available to uphold the protections for human subjects.

## Funding

This research was funded by Purdue University as part of AgSEED Crossroads or Agricultural Science and Extension for Economic Development funding to support Indiana’s Agriculture and Rural Development, the Purdue University Nutrition Education Program, and Hatch Project IND90005789. Heather Eicher-Miller received support from the Danone Institute International and the French Foundation for Medical Research as the Danone International Prize for Alimentation Laureate 2023–2024.

## Conflict of interest

The authors declare no conflict of interest. The funders had no role in the design of the study; in the collection, analyses, or interpretation of data; in the writing of the manuscript; or in the decision to publish the results.
