# Supplementary material for: BMI Was Maintained Among Women with Low Incomes in Indiana Who Participated in Food Assistance and/or Federal Nutrition Education Over 1 Year
Source: Curr Dev Nutr. 2026 Jan 30;10(3):107651. doi: 10.1016/j.cdnut.2026.107651 (PMC12954317; doi:10.1016/j.cdnut.2026.107651)
Supplement: multimedia component 1 [file mmc1.docx]

**Supplemental Table 1**. Baseline sociodemographic characteristics and comparisons by Supplemental Nutrition Assistance Program (SNAP) use among adult women Indiana Supplemental Nutrition Assistance Program Education (SNAP-Ed)-eligible participants during 2015 to 2016^a^

|  |  | SNAP participants | | SNAP nonparticipants | |  |
| --- | --- | --- | --- | --- | --- | --- |
| Characteristics |  | n | % | n | % | P-value |
| Total (n=106) |  | 67 | 63 | 39 | 37 |  |
| Age group (years) |  |  |  |  |  | 0.02* |
|  | 18-30 | 29 | 43 | 8 | 21 |  |
|  | 31-50 | 26 | 39 | 16 | 41 |  |
|  | 51 or older | 12 | 18 | 15 | 38 |  |
| Race and Ethnicity |  |  |  |  |  | 0.94 |
|  | Non-Hispanic White | 62 | 93 | 35 | 92 |  |
|  | Other | 5 | 7 | 3 | 8 |  |
| Household Education |  |  |  |  |  | 0.14 |
|  | No High School Diploma | 14 | 21 | 6 | 15 |  |
|  | High School Diploma/GED | 25 | 37 | 12 | 31 |  |
|  | Some College | 20 | 30 | 10 | 26 |  |
|  | Associate Degree | 7 | 10 | 6 | 15 |  |
|  | Bachelor’s Degree or more | 1 | 1 | 5 | 13 |  |
| Marital Status |  |  |  |  |  | 0.15 |
|  | Never Married | 16 | 24 | 5 | 13 |  |
|  | Married w/ Partner | 27 | 40 | 23 | 59 |  |
|  | Separated/Divorced/Widowed | 24 | 36 | 11 | 28 |  |
| Number of other Household Adults |  |  |  |  |  | 0.63 |
|  | None | 17 | 25 | 7 | 18 |  |
|  | 1 Additional | 24 | 36 | 17 | 44 |  |
|  | 2 Additional | 12 | 18 | 9 | 23 |  |
|  | 3 or More Additional | 14 | 21 | 6 | 15 |  |
|  |  |  |  |  |  |  |
| Number of Household Children |  |  |  |  |  | 0.26 |
|  | 0 | 15 | 22 | 14 | 36 |  |
|  | 1-2 | 29 | 43 | 16 | 41 |  |
|  | 3 or more | 23 | 34 | 9 | 23 |  |
| Employed in Last 12 Months |  |  |  |  |  | 0.59 |
|  | No | 38 | 57 | 20 | 51 |  |
|  | Yes | 29 | 43 | 19 | 49 |  |
|  | Part-Time | 17 | 59 | 7 | 39 | 0.19 |
|  | Full-Time | 12 | 41 | 11 | 61 |  |
| Other Household Adult Employment in the Last 12 Months |  |  |  |  |  | 0.85 |
|  | No | 30 | 45 | 18 | 47 |  |
|  | Yes | 36 | 55 | 20 | 53 |  |
|  | Part-Time | 7 | 20 | 6 | 32 | 0.34 |
|  | Full-Time | 28 | 80 | 13 | 68 |  |
| Monthly Income ($) |  |  |  |  |  | 0.0007*** |
|  | 0-1,265 | 30 | 45 | 7 | 18 |  |
|  | 1,266-1,705 | 16 | 24 | 5 | 13 |  |
|  | 1,706-2,144 | 10 | 15 | 7 | 18 |  |
|  | 2,145 and above | 10 | 15 | 19 | 50 |  |
| Supplemental Nutrition Assistance Program-Education |  |  |  |  |  | 0.60 |
|  | Control | 31 | 46 | 16 | 41 |  |
|  | Intervention | 36 | 54 | 23 | 59 |  |
| WIC^b^ |  |  |  |  |  | 0.20 |
|  | Yes | 29 | 43 | 12 | 31 |  |
|  | No | 38 | 57 | 27 | 70 |  |
| Emergency Food Assistance Participation (Food Pantry) |  |  |  |  |  | 0.83 |
|  | No | 32 | 48 | 19 | 50 |  |
|  | Yes | 35 | 52 | 19 | 50 |  |
|  | Less Than Once Per Month | 6 | 17 | 2 | 11 | 0.75 |
|  | One Time Per Month | 17 | 49 | 11 | 58 |  |
|  | 1-3 Times a Month | 10 | 29 | 4 | 21 |  |
|  | One Time or More Per Week | 2 | 6 | 2 | 11 |  |

GED, General Educational Development Test; WIC = Special Supplemental Nutrition Program for Women, Infants, and Children.

^a^ Data were number of participants and percent. Chi-Square tests were used to compare the characteristics. Statistical significance at P< 0.05. All data were self-reported. Total numbers do not always add up to sample size because of missing values; percentages do not always add up to 100 because of rounding.

^b^ WIC participation reference time period was the previous 30 days.

*P<0.05, **P<0.01, ***P<0.001.

**Supplemental Table 2.** Baseline sociodemographic characteristics and comparisons by Supplemental Nutrition Assistance Program (SNAP) only users and SNAP+ Special Supplemental Nutrition Program for Women (WIC) users among adult women Indiana Supplemental Nutrition Assistance Program Education (SNAP-Ed)-eligible participants during 2015 to 2016^a^

|  |  | SNAP only | | SNAP +WIC | |  |
| --- | --- | --- | --- | --- | --- | --- |
| Characteristics |  | n | % | n | % | P-value |
| Total (n=67) |  | 38 | 57 | 29 | 43 |  |
| Age group (years) |  |  |  |  |  | <0.001*** |
|  | 18-30 | 8 | 21 | 21 | 72 |  |
|  | 31-50 | 18 | 47 | 8 | 28 |  |
|  | 51 or older | 12 | 32 | 0 | 0 |  |
| Race and Ethnicity |  |  |  |  |  | 0.43 |
|  | Non-Hispanic White | 36 | 95 | 26 | 90 |  |
|  | Other | 2 | 5 | 3 | 10 |  |
| Household Education |  |  |  |  |  | 0.80 |
|  | No High School Diploma | 7 | 18 | 7 | 24 |  |
|  | High School Diploma/GED | 14 | 37 | 11 | 38 |  |
|  | Some College | 11 | 29 | 9 | 31 |  |
|  | Associate Degree | 5 | 13 | 2 | 7 |  |
|  | Bachelor’s Degree or more | 1 | 3 | 0 | 0 |  |
| Marital Status |  |  |  |  |  | 0.051 |
|  | Never Married | 6 | 16 | 10 | 34 |  |
|  | Married w/ Partner | 14 | 37 | 13 | 45 |  |
|  | Separated/Divorced/Widowed | 18 | 47 | 6 | 21 |  |
| Number of other Household Adults |  |  |  |  |  | 0.70 |
|  | None | 11 | 29 | 6 | 21 |  |
|  | 1 Additional | 12 | 32 | 12 | 41 |  |
|  | 2 Additional | 6 | 16 | 6 | 21 |  |
|  | 3 or More Additional | 9 | 24 | 5 | 17 |  |
|  |  |  |  |  |  |  |
| Number of Household Children |  |  |  |  |  | <0.0001*** |
|  | 0 | 15 | 39 | 0 | 0 |  |
|  | 1-2 | 17 | 45 | 12 | 41 |  |
|  | 3 or more | 6 | 16 | 17 | 59 |  |
| Employed in Last 12 Months |  |  |  |  |  | 0.22 |
|  | No | 24 | 63 | 14 | 48 |  |
|  | Yes | 14 | 37 | 15 | 52 |  |
|  | Part-Time | 8 | 57 | 9 | 60 | 0.88 |
|  | Full-Time | 6 | 43 | 6 | 40 |  |
| Other Household Adult Employment in the Last 12 Months |  |  |  |  |  | 0.037 |
|  | No | 21 | 57 | 9 | 31 |  |
|  | Yes | 16 | 43 | 20 | 69 |  |
|  | Part-Time | 2 | 13 | 5 | 25 | 0.39 |
|  | Full-Time | 13 | 87 | 15 | 75 |  |
| Monthly Income ($) |  |  |  |  |  | 0.30 |
|  | 0-1,265 | 19 | 50 | 11 | 39 |  |
|  | 1,266-1,705 | 10 | 26 | 6 | 21 |  |
|  | 1,706-2,144 | 6 | 16 | 4 | 14 |  |
|  | 2,145 and above | 3 | 8 | 7 | 25 |  |
| Supplemental Nutrition Assistance Program-Education |  |  |  |  |  | 0.48 |
|  | Control | 19 | 50 | 12 | 41 |  |
|  | Intervention | 19 | 50 | 17 | 59 |  |
| Emergency Food Assistance Participation (Food Pantry) |  |  |  |  |  | 0.041* |
|  | No | 14 | 37 | 18 | 62 |  |
|  | Yes | 24 | 63 | 11 | 38 |  |
|  | Less Than Once Per Month | 4 | 17 | 2 | 18 | 0.79 |
|  | One Time Per Month | 11 | 46 | 6 | 55 |  |
|  | 1-3 Times a Month | 7 | 29 | 3 | 27 |  |
|  | One Time or More Per Week | 2 | 8 | 0 | 0 |  |

GED, General Educational Development Test.

^a^ Data were number of participants and percent. Chi-Square tests were used to compare the characteristics. Statistical significance at P< 0.05. All data were self-reported. Total numbers do not always add up to sample size because of missing values; percentages do not always add up to 100 because of rounding.

*P<0.05, **P<0.01, ***P<0.001.

**Supplemental Table 3.** Baseline sociodemographic characteristics and comparisons between any food assistance program (Supplemental Nutrition Assistance Program, SNAP and/or Special Supplemental Nutrition Program for Women, Infants, and Children, WIC) participation with Supplemental Nutrition Assistance Program-Education (SNAP-Ed) intervention and no food assistance program participation and/or no SNAP-Ed intervention among adult women Indiana SNAP-Ed-eligible participants during 2015 to 2016^a^

|  |  | Group 1^b^ | | Group 2^c^ | |  |
| --- | --- | --- | --- | --- | --- | --- |
| Characteristics |  | n | % | n | % | P-value |
| Total (n=102) |  | 41 | 39 | 61 | 63 |  |
| Age group (years) |  |  |  |  |  | 0.18 |
|  | 18-30 | 18 | 4 | 19 | 29 |  |
|  | 31-50 | 12 | 29 | 30 | 46 |  |
|  | 51 or older | 11 | 27 | 16 | 25 |  |
| Race and Ethnicity |  |  |  |  |  | 0.51 |
|  | Non-Hispanic White | 37 | 90 | 60 | 94 |  |
|  | Other | 4 | 10 | 4 | 6 |  |
| Household Education |  |  |  |  |  | 0.55 |
|  | No High School Diploma | 6 | 15 | 14 | 22 |  |
|  | High School Diploma/GED | 17 | 41 | 20 | 31 |  |
|  | Some College | 11 | 27 | 19 | 29 |  |
|  | Associate Degree | 6 | 15 | 7 | 11 |  |
|  | Bachelor’s Degree or more | 1 | 2 | 5 | 8 |  |
| Marital Status |  |  |  |  |  | 0.98 |
|  | Never Married | 8 | 20 | 13 | 20 |  |
|  | Married w/ Partner | 19 | 46 | 31 | 48 |  |
|  | Separated/Divorced/Widowed | 14 | 34 | 21 | 32 |  |
| Number of other Household Adults |  |  |  |  |  | 0.33 |
|  | None | 13 | 32 | 11 | 17 |  |
|  | 1 Additional | 14 | 34 | 27 | 42 |  |
|  | 2 Additional | 8 | 20 | 13 | 20 |  |
|  | 3 or More Additional | 6 | 15 | 14 | 22 |  |
|  |  |  |  |  |  |  |
| Number of Household Children |  |  |  |  |  | 0.61 |
|  | 0 | 9 | 22 | 20 | 31 |  |
|  | 1-2 | 19 | 46 | 26 | 40 |  |
|  | 3 or more | 13 | 32 | 19 | 29 |  |
| Employed in Last 12 Months |  |  |  |  |  | 0.53 |
|  | No | 24 | 59 | 34 | 52 |  |
|  | Yes | 17 | 41 | 31 | 48 |  |
|  | Part-Time | 8 | 47 | 16 | 53 | 0.68 |
|  | Full-Time | 9 | 53 | 14 | 47 |  |
| Other Household Adult Employment in the Last 12 Months |  |  |  |  |  | 0.85 |
|  | No | 18 | 45 | 30 | 47 |  |
|  | Yes | 22 | 55 | 34 | 53 |  |
|  | Part-Time | 2 | 10 | 11 | 33 | 0.050 |
|  | Full-Time | 19 | 90 | 22 | 67 |  |
| Monthly Income ($) |  |  |  |  |  | 0.47 |
|  | 0-1,265 | 18 | 44 | 19 | 30 |  |
|  | 1,266-1,705 | 7 | 17 | 14 | 22 |  |
|  | 1,706-2,144 | 7 | 17 | 10 | 16 |  |
|  | 2,145 and above | 9 | 22 | 20 | 32 |  |
| SNAP-Ed |  |  |  |  |  | <0.0001*** |
|  | Control | 0 | 0 | 47 | 72 |  |
|  | Intervention | 41 | 100 | 18 | 28 |  |
| SNAP^d^ |  |  |  |  |  | <0.0001*** |
|  | Yes | 36 | 88 | 31 | 48 |  |
|  | No | 5 | 12 | 34 | 52 |  |
| WIC^e^ |  |  |  |  |  | 0.012* |
|  | Yes | 22 | 54 | 19 | 29 |  |
|  | No | 19 | 46 | 46 | 71 |  |
| Emergency Food Assistance Participation (Food Pantry) |  |  |  |  |  | 0.44 |
|  | No | 18 | 44 | 33 | 52 |  |
|  | Yes | 23 | 56 | 31 | 48 |  |
|  | Less Than Once Per Month | 3 | 13 | 5 | 16 | 0.59 |
|  | One Time Per Month | 11 | 48 | 17 | 55 |  |
|  | 1-3 Times a Month | 6 | 26 | 8 | 26 |  |
|  | One Time or More Per Week | 3 | 13 | 1 | 3 |  |

GED, General Educational Development Test; SNAP = Supplemental Nutrition Assistance Program; WIC = Special Supplemental Nutrition Program for Women, Infants, and Children.

^a^ Data were number of participants and percent. Chi-Square tests were used to compare the characteristics. Statistical significance at P<0.05. All data were self-reported. Total numbers do not always add up to sample size because of missing values; percentages do not always add up to 100 because of rounding.

^b^ Group 1: SNAP-Ed with SNAP and/or WIC.

^c^ Group 2: no SNAP-Ed with SNAP and/or WIC; or SNAP-Ed with neither SNAP nor WIC.

^d^ SNAP participation reference time period was the previous 30 days.

^e^ WIC participation reference time period was the previous 30 days.

*P<0.05, **P<0.01, ***P<0.001

**Supplemental Table 4**. Comparisons of baseline sociodemographic characteristics of adult Indiana Supplemental Nutrition Assistance Program-Education (SNAP-Ed)-eligible female participants to participants who withdrew from the study from 2015 to 2017^a^

|  |  | Study Sample  n =106 | | Withdrawal  n =142 | |  |
| --- | --- | --- | --- | --- | --- | --- |
| Characteristics |  | n | % | n | % | P-value |
| Age group (years) |  |  |  |  |  | 0.007** |
|  | 18-30 | 37 | 35 | 7 | 53 |  |
|  | 31-50 | 42 | 40 | 7 | 33 |  |
|  | 51 or older | 27 | 25 | 19 | 13 |  |
| Race and Ethnicity |  |  |  |  |  | 0.06 |
|  | Non-Hispanic White | 97 | 92 | 120 | 85 |  |
|  | Other | 8 | 8 | 22 | 15 |  |
| Household Education |  |  |  |  |  | 0.46 |
|  | No High School Diploma | 20 | 19 | 25 | 18 |  |
|  | High School Diploma/GED | 37 | 35 | 65 | 46 |  |
|  | Some College | 30 | 28 | 35 | 25 |  |
|  | Associate Degree | 13 | 12 | 11 | 8 |  |
|  | Bachelor’s Degree or more | 6 | 6 | 6 | 4 |  |
| Marital Status |  |  |  |  |  | 0.03* |
|  | Never Married | 21 | 20 | 50 | 35 |  |
|  | Married w/ Partner | 50 | 47 | 53 | 37 |  |
|  | Separated/Divorced/Widowed | 35 | 33 | 39 | 27 |  |
| Number of other Household Adults |  |  |  |  |  | 0.94 |
|  | None | 24 | 23 | 31 | 22 |  |
|  | 1 Additional | 41 | 39 | 59 | 42 |  |
|  | 2 Additional | 21 | 20 | 29 | 20 |  |
|  | 3 or More Additional | 20 | 19 | 23 | 16 |  |
|  |  |  |  |  |  |  |
| Number of Household Children |  |  |  |  |  | 0.37 |
|  | 0 | 29 | 27 | 34 | 24 |  |
|  | 1-2 | 45 | 42 | 73 | 51 |  |
|  | 3 or more | 32 | 30 | 35 | 25 |  |
| Employed in Last 12 Months |  |  |  |  |  | 0.003** |
|  | Yes | 48 | 45 | 91 | 64 |  |
|  | No | 58 | 55 | 51 | 36 |  |
| Other Household Adult Employment in the Last 12 Months |  |  |  |  |  | 0.77 |
|  | Yes | 56 | 54 | 78 | 56 |  |
|  | No | 48 | 46 | 62 | 44 |  |
| Monthly Income ($) |  |  |  |  |  | 0.41 |
|  | 0-1,265 | 37 | 36 | 58 | 41 |  |
|  | 1,266-1,705 | 21 | 20 | 36 | 25 |  |
|  | 1,706-2,144 | 17 | 16 | 17 | 12 |  |
|  | 2,145 and above | 29 | 28 | 31 | 22 |  |
| SNAP^b^ Participation |  |  |  |  |  | 0.23 |
|  | Yes | 67 | 63 | 79 | 56 |  |
|  | No | 39 | 37 | 63 | 44 |  |
| WIC^c^ |  |  |  |  |  | 0.036* |
|  | Yes | 41 | 39 | 74 | 52 |  |
|  | No | 65 | 61 | 68 | 48 |  |
| Emergency Food Assistance Participation (Food Pantry) |  |  |  |  |  | 0.38 |
|  | No | 54 | 51 | 81 | 57 |  |
|  | Yes | 51 | 49 | 61 | 43 |  |

GED, General Educational Development Test; SNAP = Supplemental Nutrition Assistance Program; WIC = Special Supplemental Nutrition Program for Women, Infants, and Children;

^a^ Data were number of participants and percent. Chi-Square tests were used to compare the characteristics. Statistical significance at P< 0.05. All data were self-reported. Total numbers do not always add up to sample size because of missing values; percentages do not always add up to 100 because of rounding.

^b^ SNAP participation reference time period was the previous 30 days.

^c^ WIC participation reference time period was the previous 30 days.

*P<0.05, **P<0.01, ***P<0.001
